# Influence of Coronal Flaring on the Shaping Ability of Two Heat-Treated Nickel-Titanium Endodontic Files: A Micro-Computed Tomographic Study

**DOI:** 10.3390/jcm12010357

**Published:** 2023-01-02

**Authors:** Nadine Hawi, Eugenio Pedullà, Giusy Rita Maria La Rosa, Gianluca Conte, Walid Nehme, Prasanna Neelakantan

**Affiliations:** 1 Endodontic Department, Saint Joseph University of Beirut, Rue de Damas, P.O. Box 17-5208, Beirut 1104 2020, Lebanon; 2 Department of General Surgery and Medical-Surgical Specialties, University of Catania, Via Santa Sofia 78, 95123 Catania, Italy; 3 Discipline of Endodontology, Faculty of Dentistry, The University of Hong Kong, The Prince Philip Dental Hospital, 34 Hospital Road, Hong Kong, China

**Keywords:** coronal flaring, canal transportation, centering ability, HyFlex CM, 2Shape

## Abstract

Nickel-titanium (NiTi) usage is associated in endodontics with some complications including canal transportation. Centering ability of a NiTi file is the ability to stay centered in the root canal system during instrumentation. Any undesirable deviation from the natural canal path is indicated as canal transportation. A possible strategy to improve the centering ability of NiTi instruments is the pre-enlargement of the coronal third of the root canal to minimize coronal interferences. This procedure is known as coronal flaring. The aim of this study was to perform a micro-computed tomographic (micro-CT) evaluation of the effect of coronal flaring on canal transportation and centering ability of two heat treated nickel-titanium rotary instruments, 2Shape (Micro Mega, Besançon, France) and HyFlex CM (Coltène Whaledent, Altstätten, Switzerland). Thirty extracted mandibular molars with two independent mesial canals were selected and randomly instrumented (*n* = 15 canals) with One Flare (Micro Mega, Besançon, France) before HyFlex CM, HyFlex CM (without coronal flaring), One Flare before 2Shape and 2Shape (without coronal flaring). One Flare (Micro Mega, Besançon, France) was introduced 4 mm below the canal entrance for canals prepared with coronal flaring. HyFlex CM and 2Shape were used accordingly to manufacturers’ instructions. New files were used for each canal. During and after instrumentation, irrigation procedures were performed. Micro-CT images were obtained pre- and post-preparation to measure and record root canal transportation and centralization. They were reconstructed from root apex to canal orifices, generating approximately 1000 sections per specimen. The anatomical thirds were determined by dividing the number of cross-sectional slices by three. Root canal transportation and centralization were determined by Gambil method, and the mean values were analyzed by repeated measures analysis of variance followed by multiple comparisons of Bonferroni to compare the different instrumentations procedures and the root thirds (*p* < 0.05). As for root canal transportation, 2Shape reported significantly higher values compared to HyFlex CM in the cervical region independently from the coronal flaring. In the apical region, 2Shape caused significantly minor canal transportation when used with coronal flaring with compared with the absence of coronal flaring. Regarding the centralization, HyFlex CM showed higher values than 2Shape in the cervical, independently from coronal flaring. In the apical region, 2Shape with coronal flaring exhibited significant major centering ratio, compared with not. Within the limitations of this study, coronal flaring reduced canal transportation and improved centralization of the 2Shape files in the apical section while it had no significant influence on shaping ability of the HyFlex CM instruments. Coronal flaring could represent a valid strategy to improve the shaping ability of NiTi files knowing that its benefit could be influenced by the shaping file used.

## 1. Introduction

An adequate endodontic treatment requires both mechanical and biological approach to increase the irrigants’ action and avoid bacteria recolonization [1,2]. Nickel-titanium (NiTi) rotary instruments simplified the canal preparation procedure, increasing the predictability and effectiveness of endodontic treatments [3]. Therefore, one of the main objectives of the proposed instrument designs is to reduce canal transportation and improve instrument centralization [4] through the heat treatment of NiTi alloys [5]. Heat treatments modify the austenite finish temperature (Af) of NiTi alloy, allowing the use of more flexible files at intracanal temperature with potential increase in flexural resistance [5].

The HyFlex CM (Coltène Whaledent, Altstätten, Switzerland) is a NiTi rotary instrument manufactured from a controlled-memory wire (i.e., CM-Wire) which allows to follow the anatomy of the canal, reducing the risk of ledging, transportation, and perforation [6]. The HyFlex CM 25/0.04 has a quadrangular cross-section, and the HyFlex CM 25/0.06 a triangular [6]. The 2Shape system (Micro Mega, Besançon, France) includes two rotary shaping files with an asymmetric triple helical cross-section. These instruments have been heat-treated using the T-wire technology that is claimed to enhance flexibility and ensure respect of the original root canal anatomy [7]. Shaping ability of these heat-treated files has been previously investigated demonstrating how both files ensure a predictable and safe root canal preparation [8,9].

Another strategy to improve the shaping ability of files is minimizing the anatomical interferences which could favor ledge creation or canal transportation. Particularly, coronal flaring consists in eliminating coronal interferences before apical instrumentation by the pre-enlargement of the coronal third of the root canal [10]. The coronal pre-enlargement through dedicated files aims to reduce the percentage of contact between the shaping files and the dentin walls to obtain a straighter access of the instruments to the middle and apical thirds of the canal. This further step should prevent an excessive file engagement in the dentinal walls, which normally causes torsional stress and can lead to the instrument separation [11]. Yet, preflaring may affect the residual dentin thickness and fragility of the furcation zone [12]. Currently, limited knowledge is available on the effects of coronal flaring on the behavior of shaping instruments [13]. The study of Alqahtani and AbuMostafa [13] evaluated the effect of coronal flaring and glide path on the centering ability and transportation on curved canals prepared by two NiTi files. They reported that coronal flaring and glide path did not affect the shaping ability of tested files. To the best of our knowledge, no previous studies evaluated the effects of only coronal flaring on shaping ability of heat treated NiTi files such as HyFlex CM and 2Shape rotary instruments. Thus, the aim of this study was to evaluate the influence of coronal enlargement on the root canal transportation and centralization of two rotary NiTi instruments (2Shape system and HyFlex CM) in mandibular molar mesial canals by micro-computed tomographic (micro-CT) imaging. The null hypothesis is that both systems would provide similar results in terms of centering ability and canal transportation independently from the coronal flaring.

## 2. Materials and Methods

### 2.1. Sample Selection

Sample size calculation was determined to ensure 80% power and an alpha error probability of 0.05 (G*Power 3.1.9.2 software, Heinrich-Heine-Universität Düsseldorf, Düsseldorf, Germany) attending to the results of a previous study [4]. Fifteen canals were set at the minimum sample to detect significant differences. After the approval (FMD184) by the Ethics committee of Saint Joseph University (Beirut, Lebanon), a total of 30 human mandibular molars were retained from a pool of teeth extracted for periodontal reasons and selected according to the following criteria: intact crowns without fractures, cracks, and amalgam restorations; two separate roots with completely formed apices; two separate mesial canals confirmed by periapical radiographs in a mesio-distal and bucco-lingual projection; angles of curvature between 10° and 20° measured by Schneider’s method [14] using digitized buccolingual radiographs and Sopix 2 software (Acteon, Marignac, France). Collected teeth were stored in a container with 0.1% thymol solution at 4 °C to prevent dehydration. Before shaping procedures, the samples, mounted on a custom attachment, were scanned separately using a SkyScan 1172 high-resolution X-ray microtomograph (Skyscan, Kontich, Belgium) to ensure the homogeneity of samples in terms of length, volume, and area. The scans were taken at 80 kV 100 µA, and a 4840 ms of time exposure with a 0.5 mm-thick aluminum filter. The acquired projection images were reconstructed into cross-sectional slices using the SkyScan proprietary software interface (NRecon v.1.6.4; Bruker micro-CT) with a voxel size of 12.85 µm and standardized parameters for beam hardening (30%), ring artifact correction of 10, and setting of standardized minimum and maximum contrast levels (0–0.15).

### 2.2. Sample Preparation

The specimens were randomly prepared according to the system used for root canal preparation (HyFlex CM/2Shape) with or not coronal flaring (*n* = 15):

- HyFlex CM with coronal flaring: mesial canals were flared with One Flare, then instrumented with HyFlex CM (#25/0.04; #25/0.06);
- HyFlex CM (without coronal flaring): mesial canals were instrumented with HyFlex CM without initial flaring;
- 2Shape with coronal flaring: mesial canals were flared with One Flare (#25/0.09, Micro Mega, Besançon, France), then instrumented with 2Shape (#25/0.04 and #25/0.06);
- 2Shape (without coronal flaring): mesial canals were instrumented with 2Shape without initial flaring.

Conventional access cavities were prepared using 802 diamond burs (Maillefer, Dentsply) and Endo-Z burs (Maillefer, Dentsply) with a high-speed handpiece under water cooling. After access cavity preparation, apical patency was established using a size 10 K-type file (Dentsply Syrona, Ballaigues, Switzerland) until it was visible at the apical foramen. Working length (WL) was set 1 mm shorter than the actual length of each canal. Then a glide path was established using One G (#14/0.03) (Micro Mega, Besançon, France) for all canals. One Flare was introduced 4 mm below the canal entrance for canals prepared with coronal flaring: five selective strokes on the mesial and lingual walls (for the lingual canal) or buccal (for the buccal canal) were performed.

In canals prepared with HyFlex CM, canals were shaped with HyFlex CM #25/0.04 until WL, followed by HyFlex CM #25/0.06. According to the manufacturer’s recommendations, 2.5 Ncm torque and 500 rpm speed were set on the electric motor MM Control.

In canals prepared with 2Shape, 2Shape #25/0.04 was introduced up to the WL with a progressive movement in three waves (three up-and-down movements) and an upwards filing movement. Then, 2Shape #25/0.06 was applied with the same dynamic movement until reaching the WL. Torque was set to 2.5 Ncm and speed to 300 rpm using an electric motor MM Control (Micro Mega, Besançon, France). A single expert operator performed the instrumentation. New files were used for each canal.

During shaping procedures, 5.25% sodium hypochlorite (Vista dental products; Racine, Milwaukee, USA) was intermittently deposited using a side-vented 27-G irrigation needle (Endo-Eze Irrigator, Ultradent Products, South Jordan, UT, USA) adapted to a disposable 3cc plastic syringe (Ultradent Products, South Jordan, UT, USA). After instrumentation, the canals were irrigated with 5 mL of 17% EDTA solution followed by rinsing with 3 mL distilled water. The canals were then dried with paper points and submitted to a new scan.

### 2.3. Micro-CT Analysis

**Figure 1:**
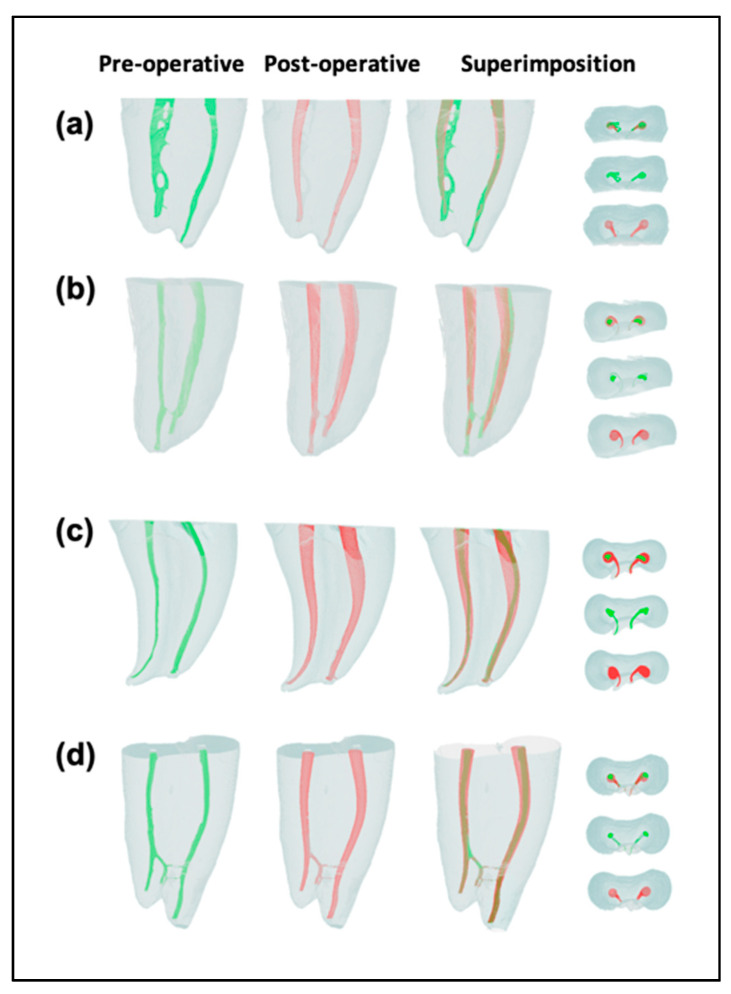
2D and 3D representations of the segmentations on mesial roots: HyFlex CM without flaring (**a**), HyFlex CM with flaring (**b**), 2Shape without flaring (**c**), 2Shape with flaring (**d**). Root canal pre-operative surfaces are indicated in green and post-operative in red. The non-instrumented canal wall surfaces were reported in green in the superposition view.

After preparation, a new micro-tomographic image was taken according to the same initial protocol to analyze the transportation and centralization of root canals. The cross-sectional images were imported into a 3D visualization and analyses software AMIRA 5.3.2. (Mercury Computer System Chelmsford, MA, USA). The preoperative and postoperative images were superimposed (Figure 1). They were reconstructed from root apex to canal orifices, generating approximately 1000 sections per specimen. The apical, middle, and coronal thirds of the canals were determined by dividing the number of cross-sectional slices by three. The mean measurement of three layers in each third generated a single thickness value of mesial and distal dentin wall for each canal before and after instrumentation [4]. All measurements were made by a blinded operator with the line measuring tool of AMIRA 5.3.2 (Mercury Computer System Chelmsford, MA, USA).

### 2.4. Root Canal Transportation

The technique developed by Gambil et al. [15] was used to measure the degree of canal transportation for all canals at the apical, middle, and cervical thirds, applying the following formula: (a1–a2)–(b1–b2); where: a1 = thinnest mesial dentin wall pre-instrumentation, a2 = thinnest mesial wall post-instrumentation, b1 = thinnest distal wall pre-instrumentation, b2 = thinnest distal wall thickness post-instrumentation. Results equal to 0 indicated that there was no canal transportation. Negative values represented transportation to the outer face of the root curvature, whereas positive values indicated transportation to the inner face.

### 2.5. Root Canal Centralization

To calculate mean centering ratio to determine the centralization of root canal preparation, the same references employed for the calculation of transportation were used; with the following formula: (a1–a2)/(b1–b2) where a1 is the mesial wall measured pre-instrumentation, a2 is the mesial wall measured post-instrumentation, b1 is the measurement of the distal wall pre-instrumentation, and b2 is the measurement of the distal wall post-instrumentation [15]. The numerator for the centering ratio formula was the smaller of the two numbers (a1–a2) or (b1–b2), if these numbers were unequal. A result of 1 indicated a perfect centralization of the instrument, whereas 0 represented complete decentralization.

### 2.6. Statistical Analysis

The software SPSS (Statistical Package Software for Social Science for Windows, Version 25.0, Chicago, IL, USA) was used for the statistical analysis. Normality of variable distribution was evaluated with the Kolmogorov–Smirnov test. Analysis of variance was used to compare canal lengths to ensure the initial comparability between all canals. Repeated measures analysis of variance was used to compare canal transportation and centering ratio between the canals for each third. The Bonferroni *t*-test multiple comparison was applied to investigate which mean values differed from one another with significance levels of *p* < 0.05.

## 3. Results

### 3.1. Root Canal Transportation

Preoperative root canal lengths, volumes, and areas were not significantly different between the all canals (*p* > 0.05); therefore, specimens were comparable.

**Figure 2:**
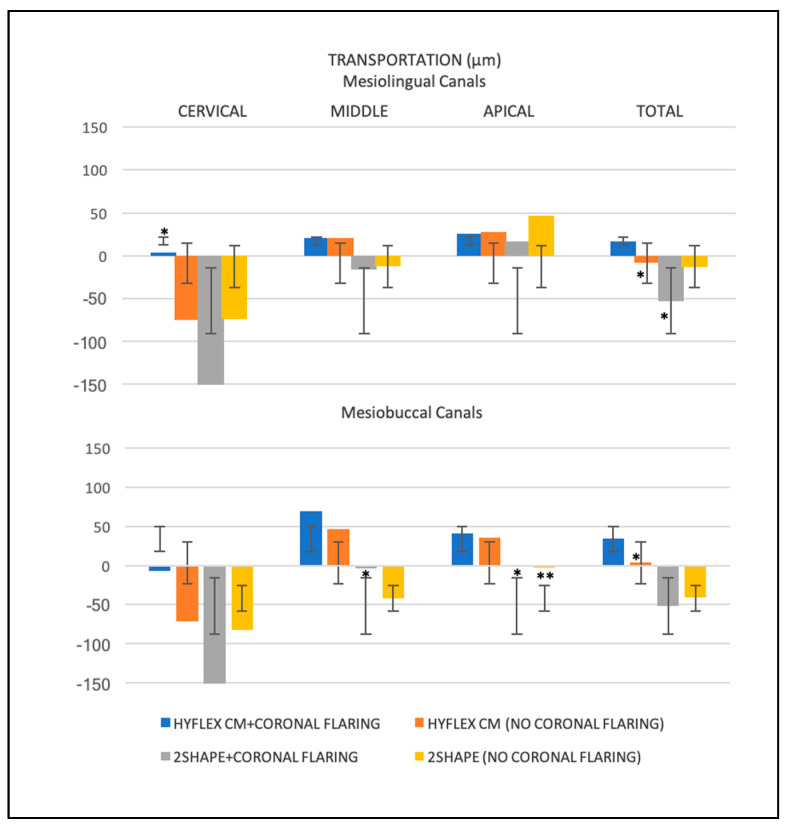
Histogram of the mean values (µm, micrometers) and standard deviations (I) of the root canal transportation for each instrumentation procedure at different anatomical thirds. *, ** indicate a statistically significant difference between the different instrumentation procedures for the same anatomical third.

The mesial canal transportation results and the differences between mesiolingual and mesiobuccal canals are presented in Table 1 and Figure 2, respectively.

Considering each anatomical third, 2Shape reported significantly higher values compared to HyFlex CM in the cervical region (*p* < 0.05), independently from the coronal flaring (*p* > 0.05), while no significant differences emerged in the middle thirds between all instrumentation procedures (*p* > 0.05). In apical, 2Shape caused significantly minor canal transportation when used with coronal flaring with respect to not (*p* < 0.05). The other comparisons were not statistically significant (*p* > 0.05).

No significant differences emerged between the different root canal thirds for each instrumentation procedure except for 2Shape for which coronal region exhibited more canal transportation (*p* < 0.05), independently from coronal flaring (*p*>0.05).

Finally, HyFlex CM with no coronal flaring showed the lowest total canal transportation in both mesiolingual and mesiobuccal canals (*p*<0.05).

### 3.2. Root Canal Centralization

**Figure 3:**
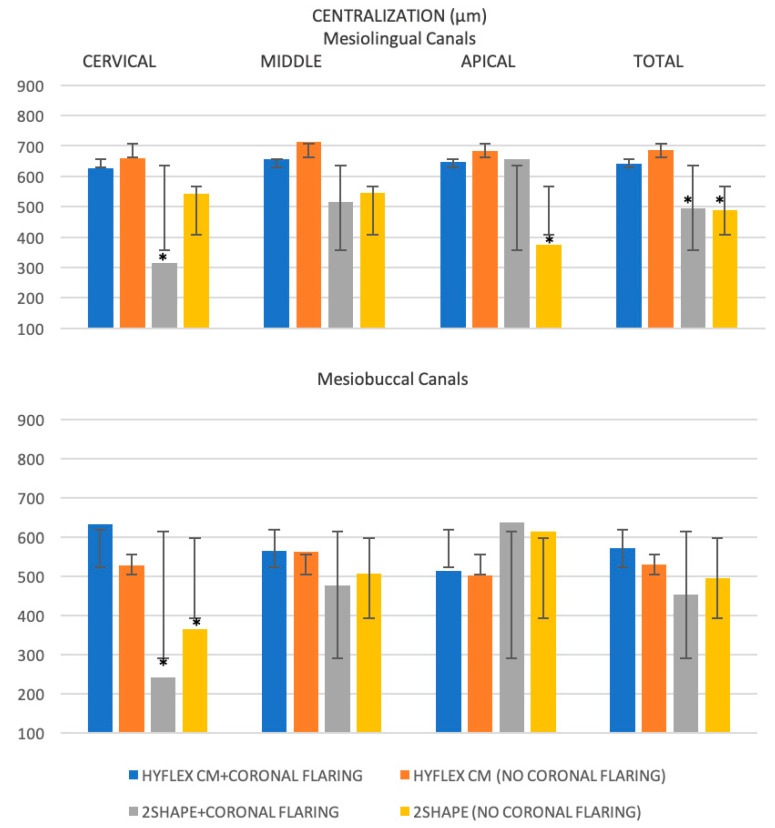
Histogram of the mean values (µm, micrometers) and standard deviations (I) of the root canal centralization for each instrumentation procedure at different anatomical thirds. * indicate a statistically significant difference between the different instrumentation procedures for the same anatomical third.

The centralization results obtained for the mesial canals and the differences between mesiobuccal and mesiolingual canals are shown in Table 2 and Figure 3, respectively.

Considering the anatomical thirds, the centralization values were significantly higher for HyFlex CM compared with 2Shape in the cervical (*p* < 0.05), independently from coronal flaring (*p* > 0.05) while no significant differences emerged in the middle thirds (*p* > 0.05). As concerns for apical region, 2Shape with coronal flaring exhibited significant major centering ratio compared with not (*p* < 0.05).

No significant differences emerged between the different root canal thirds for each instrumentation procedure (*p* > 0.05).

Finally, the HyFlex CM with or without coronal flaring showed the significant major total centering ratio in the mesiolingual canals (*p* < 0.05), while no significant difference emerged between the all instrumentation procedures in the mesiobuccal canals (*p* > 0.05).

## 4. Discussion

The present study aimed to evaluate the effects of coronal preflaring on canal transportation and centralization of root canal preparation of 2Shape and HyFlex CM rotary files. Coronal flaring was performed with One Flare and the glide path with One G for all canals to standardize the procedure. As previously reported [4,16], mesial roots were selected because they often present a triangle of dentin that needs to be removed in order to have a more direct access to the apical area [17]. Different techniques have been used to evaluate transportation such as the sectional technique [18] which enabled direct visualization of canal deviation. However, it required a long tooth preparation with loss and alteration of dental tissue, determining less precise results. The superimposing technique of conventional preoperative and postoperative radiographs was also used [16,19], but it provided an inaccurate and unreliable two-dimensional image. Conversely, micro-CT is considered as the reference three-dimensional radiological method in dental research, providing reliable findings for clinical applications [4,18]. The 2Shape and HyFlex CM have been selected because they are commonly used in clinical practice and are easily comparable because of the same dimensions.

According to the present results, coronal flaring had no significant effect on the HyFlex CM while it improved shaping ability of 2Shape files in the apical region. Thus, the null hypothesis can be partially rejected.

The centering ability of an instrument is affected by several factors [20], including the alloy properties [20,21]. The traditional microstructure of T-wire alloy is modified and its Af (austenite finish) temperature is lower than body temperature, thus remaining in the austenitic phase [22,23]. Conversely, the HyFlex CM instruments have undergone a different thermomechanical process, which allows to achieve an Af temperature of approximately (50 °C) [24]. Thus, the behavior of HyFlex CM files may be explained by the presence of stable martensite phase at temperatures below the Af. In addition, these instruments—using the CM-wire technology—have no rebound effect after unloading [25] reducing canal transportation compared to the other instruments, in agreement with some previous studies [8,26,27]. Moreover, the heat treatment reduces the hardness of the alloy and consequently its cutting efficiency [28], with a lower risk of canal deviation [29]. Thus, it is plausible to hypothesize that the coronal enlargement could advantage most of the stiffer files, such as 2Shape, allowing them to achieve a better centralization in the apical region. Of note, independently from coronal flaring, all values obtained for apical transportation were less than 0.15 mm, which is considered the acceptable limit [30,31].

No previous studies determined the effects of coronal flaring on shaping ability of HyFlex CM and 2Shape, thus a direct comparison with our results is not possible.

Regarding the effect of coronal flaring on shaping ability of NiTi files, a previous study of Barbieri et al. [16] found no difference between pre-flared and not pre-flared canals when using Reciproc and Waveone. The differences with our study are probably caused by the different kinds of instrument tested and evaluation system (i.e., the superimposition of radiographs vs. micro-CT).

Considering mesiolingual and mesiobuccal canals, HyFlex CM (without coronal flaring) presented the lowest values of canal transportation in both canals even if the transportation occurred towards the inner surface of the curvature (i.e., the mesial direction) in the mesiobuccal canal. This last situation is more clinically dangerous because it involves the cervical zone which is more susceptible to perforations due to the reduced dentinal thicknesses [32]. Regarding the centralization ability, HyFlex CM revealed major centering ratio in mesialingual canals compared to those with 2Shape, independently form coronal flaring and there was no significant difference between the instruments in mesiobuccal canals. This variation may be referred to the most complex anatomical configurations of mesiobuccal canals, which neutralizes the benefits provided by the instrumentation.

Some limitations are to be considered. First, many factors can affect shaping ability including instrument size and taper, as well as the design and type of alloy. Thus, our findings should be extended to other conditions with caution. Second, anatomical complexities need to be evaluated in clinical conditions. Further studies (in vitro and in vivo) are warranted to confirm and generalize these results. In addition, despite all procedures being performed by a single operator to minimize bias, a certain degree of variability in use of free hand instrumentation may have remained. Thus, free hand instrumentation can be a confounding factor and should be considered as another possible study limitation.

Overall, our findings support that canal transportation is major in the cervical regions of root canals. Moreover, coronal flaring could be clinically useful in increasing the centering ability, especially of the austenitic files in the apical third.

## 5. Conclusions

Within the limitations of this study, coronal flaring resulted in reduced transportation and improved centralization of 2Shape system in the apical thirds while had no effect on HyFlex CM system. The HyFlex CM was more centered and caused less canal transportation than the 2Shape in the coronal third independently from the coronal flaring probably associated with alloy properties. All tested instruments were confirmed to be relatively safe in root canal preparation and in original canal anatomy preservation.

## Figures and Tables

**Table 1:** Means (mm, millimeters) ± standard deviations of root canal transportation with the different instrumentation procedures for each root canal section.

	Root Canal Transportation (mm)			
Root Canal Thirds	HyFlex CM+Coronal Flaring	HyFlex CM (No Coronal Flaring)	2Shape+Coronal Flaring	2Shape (No Coronal Flaring)
**Cervical**	0.115 ^a^ ± 0.048	0.147 ^a^ ± 0.071	0.247 ^b,^* ± 0.115	0.216 ^b,^* ± 0.117
**Middle**	0.090 ^a^ ± 0.066	0.079 ^a^ ± 0.037	0.073 ^a^ ± 0.047	0.121 ^a^ ± 0.084
**Apical**	0.049 ^a,b^ ± 0.021	0.056 ^a,b^ ± 0.039	0.024 ^a^ ± 0.020	0.065 ^b^ ± 0.037
**Total**	0.084 ^a^ ± 0.045	0.031 ^a^ ± 0.049	0.114 ^a^ ± 0.060	0.134 ^a^ ± 0.079

^a,b^ indicate a statistically significant difference between the different instrumentation procedures in the same row. * indicate a statistically significant difference between the different root thirds in the same column.

**Table 2:** Means (mm, millimeters) ± standard deviations of root canal centralization with the different instrumentation procedures for each root canal section.

	Root Canal Centralization (mm)			
Root Canal Thirds	HyFlex CM+Coronal Flaring	HyFlex CM (No Coronal Flaring)	2Shape+Coronal Flaring	2Shape (No Coronal Flaring)
**Cervical**	0.629 ^a^ ± 0.117	0.594 ^a^ ± 0.137	0.278 ^b^ ± 0.246	0.455 ^b^ ± 0.170
**Middle**	0.612 ^a^ ± 0.104	0.639 ^a^ ± 0.185	0.496 ^a^ ± 0.192	0.527 ^a^ ± 0.184
**Apical**	0.580 ^a,b^ ± 0.112	0.593 ^a,b^ ± 0.178	0.647 ^a^ ± 0.199	0.495 ^b^ ± 0.162
**Total**	0.607 ^a^ ± 0.111	0.608 ^a^ ± 0.166	0.473 ^a^ ± 0.212	0.492 ^a^ ± 0.172

^a,b^ indicate a statistically significant difference between the different instrumentation procedures in the same row.

## Data Availability

The datasets generated during the current study are available from the corresponding author on reasonable request.
